# FNA of misclassified primary malignant neoplasms of the thyroid: Impact on clinical management

**DOI:** 10.4103/1742-6413.45191

**Published:** 2009-01-19

**Authors:** Sejal S. Shah, William C. Faquin, Roberto Izquierdo, Kamal K. Khurana

**Affiliations:** Department of Pathology, SUNY Upstate Medical University, Syracuse, NY, USA; 1 Department of Pathology, Massachusetts General Hospital, Harvard Medical School Boston, MA, USA; 2 Department of Medicine (Endocrinology), Upstate Medical University, Syracuse, NY, USA

**Keywords:** Fine needle aspiration, management, misclassified neoplasms, thyroid

## Abstract

**Background:**

Fine needle aspiration (FNA) cytology is a popular, reliable and cost effective technique for the diagnosis of thyroid lesions. The aim of our study was to review cases of misclassified primary malignant neoplasms of the thyroid by FNA, and assess the causes of cytologic misdiagnosis and their impact on clinical management.

**Methods:**

Clinical data, FNA smears and follow-up surgical specimens of cases diagnosed with primary thyroid carcinoma were reviewed.

**Results:**

Of the 365 cases with a malignant diagnosis by FNA over a period of 11 years, nine (2.4 %) were identified with discrepant histologic diagnosis with regard to the type of primary thyroid malignancy. In addition, four cases were added from the consultation files of the Massachusetts General Hospital. Areas of difficulty contributing to misclassification included overlapping cytologic features (n = 6), rarity of tumors (n = 3), and sampling limitations (n = 4). Of the 13 cases, 12 underwent total or near total thyroidectomy and one patient had concurrent surgical biopsy. Measurement of serum calcitonin levels in one case, with an initial cytologic diagnosis of medullary carcinoma, prevented unnecessary lymph node dissection. Misclassification of medullary carcinoma as papillary carcinoma precluded lymph node dissection in one case. Further management decisions were based on the final histologic diagnosis and did not require additional surgery. Two cases of undifferentiated (anaplastic) thyroid carcinoma were misdiagnosed as papillary thyroid carcinoma. Both patients received total thyroidectomies, which may not otherwise have been performed.

**Conclusions:**

A small subset of primary malignant neoplasms of the thyroid may be misclassified with regard to the type of malignancy on FNA. The majority of primary malignant neoplasms diagnosed on FNA require thyroidectomy. However, initial cytologic misclassification of medullary carcinoma or undifferentiated carcinoma as other malignant neoplasms or vice versa may have an impact on clinical management.

## BACKGROUND

Fine needle aspiration (FNA) cytology is a reliable and economical initial test for the diagnosis of thyroid nodules, both benign and malignant. The overall sensitivity of a definitive diagnosis has been reported to be 83-92%, and the specificity ranges from 75-97%.[1–5] With satisfactory specimens, the diagnostic accuracy of FNA for thyroid nodules can be as high as 95%.[2,6] Positive predictive values can range from 89% to 98%, and negative predictive values from 94% to 99%.[7]

The most common thyroid cancers diagnosed by FNA are papillary thyroid carcinoma, followed by follicular carcinoma, medullary thyroid carcinoma, undifferentiated carcinoma, and poorly differentiated carcinoma.

The management of thyroid cancer is strongly influenced by the FNA diagnosis and since clinical treatment algorithms vary depending upon the specific subtype of thyroid cancer, especially with regard to surgical intervention, an accurate subclassification of the malignancy is important. The aim of our study was to conduct a retrospective analysis of thyroid FNA cases that were misclassified, based upon the type of primary malignancy diagnosed; to identify the causes of misdiagnosis; and, to assess the impact on clinical management.

## MATERIALS AND METHODS

A search of the cytology files of the State University of New York Health Science Center; Syracuse, New York, from 1996 to 2006, was conducted. All cases with a diagnosis of primary thyroid cancer were evaluated, and cases with histologic follow-up were included in the study. Four additional cases were added from the consultation files of the Massachusetts General Hospital. All FNAs were performed using 25- or 23-gauge (0.25 or 0.23 mm) needles. In all the cases, the material obtained was smeared onto uncoated glass slides and either air-dried or fixed in ethanol for Diff-Quik or a Papanicolaou stain, respectively. When appropriate material for a cell block was procured by FNA, it was fixed in 10% neutral-buffered formalin, embedded in paraffin, sectioned at 4μ and stained with hematoxylin and eosin. The latter methods also were applied to histologic samples.

Histochemical stains like mucicarmine and immunocytochemical studies were performed in selected cases, on paraffin embedded sections by the labeled streptavidin-biotin peroxidase method. The antibodies used included those against thyroglobulin (rabbit polyclonal, 1 : 500; Dako Co., Carpenteria, CA); TTF-1 (mouse monoclonal, 1 : 30; Biogenex, San Ramon, CA); calcitonin (rabbit polyclonal, 1 : 200; Lab Vision Corp., Fremont, CA); CK19 (mouse monoclonal, 1 : 50; Fischer Scientific, Pittsburgh, PA); CEA (rabbit polyclonal, 1 : 1000; Lab Vision Corp.); chromogranin (mouse monoclonal, 1 : 300; Lab Vision Corp.); synaptophysin (mouse monoclonal, 1 : 50; Biogenex) and HBME (mouse monoclonal, 1 : 50; Dako Co.). Additional immunohistochemical stains used in the cases from the consultation files included Ki-67, p53, CD45, CD30, HMB45, CD79a, CD20, CD10, Bcl-6, Bcl-2, Pax 5, CD5, CD138, HHV-8, IgG, IgA and IgM. In situ hybridization studies for EBER, and kappa and lambda light chain were also performed.

## RESULTS

Of the 365 cases with a malignant diagnosis on FNA, over a period of 11 years, nine (2.4 %) were identified with a discrepant histologic diagnosis, with regard to the type of primary thyroid malignancy. In addition, four cases (Cases 5,6.9,11) were added from the consultation files of the Massachusetts General Hospital. Of these 13 cases, the error in misclassification of thyroid malignancy was attributed to overlap in cytologic features (n = 6), rarity of tumors (n = 3) and inadequate sampling (n = 4).

Table 1 summarizes the cytologic and histologic diagnoses, and clinical management in cases with overlapping cytologic features. Three of the six cases were diagnosed as papillary thyroid carcinoma, as they showed rare intranuclear inclusions and slight nuclear irregularity with rare nuclear grooves. One of these cases showed few plasmacytoid cells and was diagnosed as medullary carcinoma on histology, while the other two cases were Hurthle cell carcinoma and follicular carcinoma.

**Table 1:** Misclassified malignant neoplasms of the thyroid: Overlapping cytological features

*Case #*	*Age/ gender*	*Cytologic diagnosis*	*Management*	*Histologic diagnosis*	*Clinical Impact*
1	67/F	Papillary carcinoma	Near total thyroidectomy	Hurthle cell carcinoma	None
2	46/F	Papillary carcinoma	Near total thyroidectomy	Follicular carcinoma	None
3	58/M	Papillary carcinoma	Near total thyroidectomy	Medullary carcinoma	Significant
4	44/F	Medullary carcinoma	Normal Serum calcitonin. Near total thyroidectomy with lymph node excision.^a^	Papillary carcinoma	None
5	74/F	Follicular carcinoma	Total thyroidectomy	Hurthle cell carcinoma	None
6	73/F	Poorly differentiated carcinoma	Total thyroidectomy	Follicular carcinoma	None

M = Male, F = Female

a Metastatic carcinoma was identified

**Figure 1:**
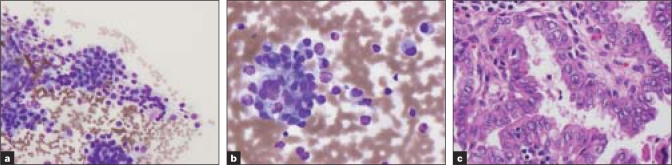
Table 1; Case 4 (a, b) Aspirate smears show single cells and cells in small clusters with eccentric small uniform nuclei with fine chromatin and abundant cytoplasm (Diff-Quik^®^ stain). (c) Histologic sections revealed papillary carcinoma (H&E)

Two other cases were misclassified as medullary carcinoma by FNA [Figure 1], due to the presence of plasmacytoid single cells with abundant cytoplasm, and follicular carcinoma due to the presence of abundant follicular cells. The excision specimen revealed papillary carcinoma and Hurthle cell carcinoma respectively. Aspirate smears of the sixth case revealed features of poorly differentiated carcinoma. Excision revealed follicular carcinoma with oncocytic features. The tumor cells also exhibited extensive degenerative changes with moderate atypia and occasional mitoses.

**Figure 2:**
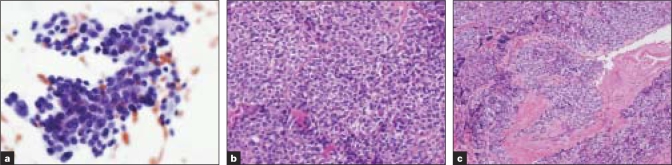
Table 2; Case 1 (a) Aspirate smears show papillary clusters of cells with nuclear grooves and rare intranuclear inclusions (Papanicolaou stain). Histologic sections (b, c) show insular carcinoma with vascular invasion (Hematoxylin and Eosin [H&E] stain)

Three cases were misclassified due to the rarity of the tumors [Table 2]. Two of these cases showed papillary clusters of cells and were misdiagnosed as papillary carcinoma; however, histology revealed insular carcinoma with extrathyroidal extension, areas of necrosis and active mitoses [Figure 2]. Aspirate smears of the third case revealed discohesive pleomorphic single cells, morphologically consistent with large cell lymphoma. Material was not available for flow cytometry or additional immunostains. Concurrent biopsy revealed large sheets of malignant cells, many with anaplastic nuclei and moderate cytoplasm. Some more differentiated plasma cells were also seen. The tumor cells were lambda light chain restricted and positive for CD45, weakly positive for CD79a and negative for CD20, CD30, CD10, CD5, BCl-2, Bcl-6, Pax5 and S100, and consistent with anaplastic plasmacytoma. In situ hybridization for EBER and immunostain for HHV-8 and heavy chains were negative.

**Table 2:** Misclassified malignant neoplasms of the thyroid: Rarity of tumors

*Case #*	*Age/ Gender*	*Cytologic diagnosis*	*Management*	*Histologic diagnosis*	*Clinical Impact*
7	58/F	Papillary carcinoma	Near total thyroidectomy. Recurrence of tumor in soft tissue and lymph nodes after 2 years	Insular carcinoma	None
8	78/F	Papillary carcinoma	Near total thyroidectomy with soft tissue and lymphnode excision.^a^	Insular carcinoma	None
9	76/F	Large cell lymphoma	Concurrent biopsy	Anaplastic plasmacytoma	None

F= Female

a Metastatic carcinoma was identified

**Figure 3:**
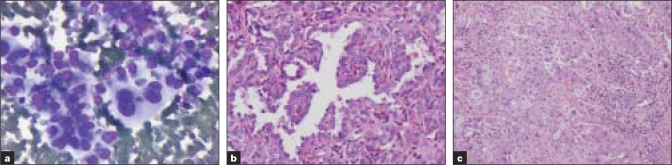
Table 3; Case 3 (a) Aspirate smears reveal pleomorphic cells with eccentric nuclei, macronucleoli and cytoplasmic vacuoles (Diff-Quik^®^ stain) that were mucicarmine positive (b) Histologic sections of the thyroid revealed papillary carcinoma and those of lymph node (c) showed papillary carcinoma with mucinous metaplasia (H&E)

Inadequate sampling was the cause of misclassification in four cases [Table 3]. Two of these showed cytological features of papillary carcinoma. The excision revealed anaplastic carcinoma with extrathyroidal extension. Focally well-differentiated papillary carcinoma with transition into anaplastic areas was seen in both cases. FNA diagnosis of the third case was adenocarcinoma, due to the presence of tumor cells with eccentric pleomorphic nuclei, macronuclei and cytoplasmic vacuolization [Figure 3], with demonstration of intracytoplasmic mucin by mucicarmine stain. Histology, however, revealed papillary carcinoma with focal solid mucin producing regions positive for CK19 and HBME and weakly positive for TTF1 and thyroglobulin.

**Table 3:** Misclassified malignant neoplasms of the thyroid: Inadequate sampling

*Case #*	*Age/ gender*	*Cytologic diagnosis*	*Management*	*Histologic diagnosis*	*Clinical impact*
10	71/M	Papillary carcinoma	Total thyroidectomy with soft tissue and lymph node excision.^a^,^b^	Anaplastic carcinoma with extrathyroidal extension	Significant
11	84/F	Papillary carcinoma	Total thyroidectomy	Anaplastic carcinoma with extrathyroidal extension	Significant
12	64/M	Adenocarcinoma	Near total thyroidectomy with lymph node excision^a^	Papillary carcinoma	None
13	82/M	Myxoid sarcoma	Near total thyroidectomy	Papillary carcinoma	None

M = Male, F= Female

a Metastatic carcinoma was identified

b Intraoperative frozen section

The cytologic findings of the fourth case have been previously reported,[8] where a diagnosis of myxoid liposarcoma was rendered. Aspirate smears revealed a monomorphic population of bland spindle cells admixed with myxoid material. Occasional spindle cells with hyperchromatic pleomorphic nuclei and nuclear atypia were also seen and a cytologic diagnosis of myxoid liposarcoma was rendered. Histologic sections revealed a tumor composed of an epithelial component typical for papillary carcinoma with a stromal component reminiscent of nodular fasciitis.

Of the 13 cases, 12 underwent total or near total thyroidectomy and one case underwent concurrent biopsy. Intraoperative findings in four cases resulted in the excision of lymph nodes, which, in the all cases, were positive for metastatic carcinoma.

## DISCUSSION

Differentiated thyroid carcinomas account for more than 90% of all thyroid cancers, and surgical treatment is the first line of management. Various scoring systems have been proposed to classify these patients into low and high risk categories, depending upon variables including patient age, metastases, extrathyroidal extension, and tumor size.[9–11] For high-risk patients, current guidelines recommend total or near total thyroidectomy.[12] In addition, patients with bilateral tumors, extrathyroidal extension, or local and distant metastasis are recommended to have a total or near total thyroidectomy. Treatment of low risk patients, however, is controversial and debated between lobectomy and total or near total thyroidectomy. Most recent studies, however, recommend total or near total thyroidectomy for all differentiated carcinomas that are larger than 1 cm.[13] The advantages of total or near total thyroidectomy include the use of radioactive iodine and serum thyroglobulin to detect and treat recurrent carcinoma and metastatic disease and to eliminate contralateral occult cancers.[14] Further, studies have shown that patients with total or near total thyroidectomy have decreased rates of recurrence and improved survival.[15] Lymph node excision is recommended for papillary and follicular carcinoma, when they appear enlarged during intraoperative assessment or by ultrasonography.

The recommended treatment of medullary carcinoma includes total thyroidectomy, central compartment lymph node dissection and ipsilateral (unilateral) modified radical neck dissection, as these have a high rate of recurrence.[12] In contrast to well- and poorly differentiated thyroid carcinomas and medullary thyroid carcinoma, anaplastic thyroid carcinomas, unless small and limited to the thyroid gland, are not managed surgically.

A majority of our cases [Table 1] were misclassified due to overlap in cytological features. Papillary thyroid carcinomas cytologically produce cellular aspirate smears with papillary structures and nuclear features which include nuclear irregularity and grooving and nuclear inclusions. However, these rarely may be present in other benign and malignant conditions including medullary carcinoma, follicular carcinoma and Hurthle cell carcinoma and hence caution should be exercised, especially when these features are present focally.[16–19] Although demonstration of calcitonin and CEA positive tumor cells using immunohistochemical stains can further support the diagnosis of medullary carcinoma, negative staining does not exclude the diagnosis. In our case of papillary carcinoma, misdiagnosed as medullary carcinoma, diagnostic material was noted on single slide and no cell block material was available. Immunohistochemical studies were not performed, since we did not want to compromise the diagnostic material. The diagnosis of follicular carcinoma on cytology is not possible due to the inability to recognize capsular invasion on cytology smears. However, tumors presenting with overt malignant features may be classified as poorly differentiated carcinoma and may represent follicular carcinoma as noted in case10. In case 9 with histologic diagnosis of Hurthle cell carcinoma, the granular features of cytoplasm of malignant cells were not as overt in cytology smears, as seen in histologic sections. Distinction between Hurthle cell carcinoma and follicular carcinoma may be more of a semantic issue.

In our study, primary tumor misclassification could be attributed to the rarity of tumors in three cases [Table 2]. Two of these cases were insular carcinomas, which were misclassified as papillary thyroid carcinomas. These are uncommon primary thyroid tumors and their cytological features, though not well defined, include cellular aspirates with mostly single cells with round monomorphic nuclei. The cells may also be present in sheets and may show microfollicles. These tumors can also show focal nuclear grooves and intranuclear pseudoinclusions.[20] In case 13, anaplastic plasmacytoma was classified as large cell lymphoma on aspirate smears. To our knowledge, anaplastic plasmacytoma of the thyroid has not been previously described. Additional material available for surgical biopsy allowed for further work with immunostains, which supported the diagnosis of anaplastic plasmacytoma. The distinction between anaplastic plasmacytoma and large cell lymphoma may have important clinical implications.[21] Extramedullary solitary plasmacytoma may experience prolonged survival with simple local excision or radiotherapy, whereas large cell lymphoma may have aggressive course and require combined chemotherapy to ensure favorable results.[21]

In our study, four cases [Table 3] were misclassified due to inadequate sampling. These included two cases of undifferentiated carcinoma (anaplastic carcinoma), where FNA sampling was limited to areas with predominately papillary carcinoma like cytologic features. Anaplastic thyroid carcinoma usually presents in the seventh decade of life.[22] Clinical findings include rapidly growing mass, loss of weight, hoarseness and fixed mass in the neck and multiple lymph node involvement and dysphagia.[22] In both our cases, patients presented with enlarged solid thyroid nodule and hoarseness, findings that may also be seen in case of papillary carcinoma. Hence, in our cases, the clinical findings did not help in distinguishing anaplastic carcinoma from papillary carcinoma. FNA smears in case 12 revealed tumor cells with mucinous material and areas typical for papillary carcinoma were not sampled. Mucinous metaplasia of papillary thyroid carcinoma, as seen in case 11, is extremely uncommon but a potential pitfall in the cytopathology of aspiration smears.[23,24] Similarly, our case 13 of papillary carcinoma, with nodular fasciitis like stroma (case 12), also posed difficulty on FNA, due to sampling areas being limited to areas of nodular fascitis like stroma, which was misinterpreted as myxoid sarcoma.[8] Although sampling different areas of lesion may yield better cytologic sample, sampling limitation in fine needle aspiration can be a source of misinterpretation, as noted in our cases.

Of the 13 cases in our study, 12 underwent total or near total thyroidectomy and one patient had concurrent surgical biopsy. In one of our cases that were misclassified as medullary carcinoma, the measurement of serum calcitonin levels precluded the unnecessary lymph node excision in the case. Elevated serum calcitonin in the presence of a thyroid nodule is characteristic of medullary carcinoma and routine basal serum calcitonin levels has been recommended to be considered as an integral part in the diagnostic evaluation of thyroid nodules.[25,26]

The most significant clinical impact on management was in a case of medullary carcinoma that was misclassified as papillary carcinoma on initial cytologic evaluation and in two cases of anaplastic carcinoma that were misclassified as papillary carcinoma. The patient with medullary carcinoma was treated with near total thyroidectomy without central compartment lymph node dissection. However, at five and a half years of follow up, this patient has had no additional surgery or recurrences. Both cases of anaplastic carcinoma were treated with total thyroidectomy and, in addition, neck lymph node excision was performed in one case. Extrathryoidal extension was noted in both the cases. With preoperative diagnosis of anaplastic carcinoma in these patients, surgical treatment could have been avoided. At last follow up visits at 3 months and 6 months, both patients were alive.

In conclusion, a small fraction of primary malignant neoplasms of the thyroid may be misclassified with regard to the type of malignancy on FNA. The majority of primary malignant neoplasms diagnosed on FNA require thyroidectomy. However, initial cytologic misclassification of medullary carcinoma or anaplastic carcinoma as other malignant neoplasms or vice versa may have an impact on the decision to perform a central compartment lymph node dissection for medullary thyroid cancer or thyroidectomy at all for anaplastic thyroid carcinoma

## COMPETING INTEREST STATEMENT BY ALL AUTHORS

No competing interest to declare by any of the authors.

## AUTHORSHIP STATEMENT BY ALL AUTHORS

Each author acknowledges that this final version was read and approved.

According to International Committee of Medical Journal Editors (ICMJE http://www.icmje.org) “author” is generally considered to be someone who has made substantive intellectual contributions to a published study.

Authorship credit should be based on 1) substantial contributions to conception and design, acquisition of data, or analysis and interpretation of data; 2) drafting the article or revising it critically for important intellectual content; and 3) final approval of the version to be published. Authors should meet conditions 1, 2, and 3. Other contributors, who do not meet these criteria for authorship, are listed in an acknowledgments section.

All authors of this article declare that we qualify for authorship as defined by ICMJE http://www.icmje.org/#author.

Each author has participated sufficiently in the work and take public responsibility for appropriate portions of the content of this article.

## ETHICS STATEMENT BY ALL AUTHORS

This study was conducted with approval from Institutional Review Board (IRB) (or its equivalent) of all the institutions associated with this study.

Authors take responsibility to maintain relevant documentation in this respect.
